# Geometrical analysis of the BeFlared bridging stent-graft in fenestrated endovascular aneurysm repair

**DOI:** 10.1186/s42155-026-00670-2

**Published:** 2026-03-28

**Authors:** Natasha Hasemaki, Zicheng Wan, Jan Stana, Alexia-Vasiliki Amvrazi, Baban Assaf, David Khangholi, Nikolaos Konstantinou, Nikolaos Tsilimparis

**Affiliations:** 1 Department of Vascular Surgery, University Hospital LMU Munich, Munich, Germany; 2 Department of Vascular Surgery, the Second Xiangya Hospital of Central South University, Changsha, China

**Keywords:** Fenestrated endovascular aortic repair, Complex abdominal aortic endovascular repair, Bridging stent, Flaring technique

## Abstract

**Objective:**

To evaluate the geometrical configuration of the BeFlared bridging stent-graft and the early clinical outcomes in fenestrated endovascular aneurysm repair (FEVAR) for complex abdominal and thoracoabdominal aortic aneurysms.

**Methods:**

This retrospective single-center study included 23 consecutive patients who underwent FEVAR using at least one BeFlared stent-graft between December 2024 and June 2025. Technical and clinical data were collected prospectively. Geometrical analysis of the stent-graft configuration was performed using postoperative computed tomography angiography (CTA) at 1 month.

**Results:**

A total of 70 target vessels (TVs) were treated with BeFlared stent-grafts, achieving a 100% technical success rate. No TV-related endoleaks, instability, or reinterventions were observed at 30-day follow-up. Geometric analysis demonstrated a mean protrusion length of 5.27 ± 0.87 mm and a flare ratio of 0.96 ± .03. The mean protrusion length was 5.4 ± .12 mm for renal stent-grafts and 5.1 ± .18 mm for visceral stent-grafts (*p* = .155). The mean flare ratio was higher in renal stent-grafts (0.97 ± .03) compared to visceral stent-grafts (0.95 ± .03), (*p* = .045), though both were within optimal ranges. The mean opening flaring angle was 42.3° ± 19.1 for renal stent-grafts and 41.2° ± 19.4 for visceral stent-grafts (*p* = .809).

**Conclusion:**

Early clinical and technical outcomes of the BeFlared stent-graft in FEVAR are promising, with satisfactory procedural performance and favorable geometric configuration. Larger, multicenter studies with longer follow-up are essential to confirm long-term durability and clinical benefit.

## Introduction

Fenestrated endovascular aneurysm repair (FEVAR) has become the first-line treatment strategy in most centers for patients with complex abdominal aortic aneurysms (cAAAs) and thoracoabdominal aortic aneurysms (TAAAs) who have suitable anatomy [1, 2]. With the continued development and use of physician-modified endografts (PMEGs) [3] an even larger proportion of patients with cAAAs can now be treated—particularly in regions where custom-made devices (CMDs) are not yet approved or when urgent clinical scenarios necessitate immediate intervention. Most published series from high-volume centers show favorable mid-term clinical outcomes and high technical success rates for both CMDs and PMEGs [3–6].

Over the past years, technological advances in the main endograft design, as well as the use of fusion technology and the development of adjuvant endovascular tools (steerable sheaths, intravascular lithotripsy, etc.) have facilitated the broad applicability and feasibility of fenestrated techniques either via custom-made or PMEGs [7–9]. However, until recently a major drawback of the fenestrated technique was the absence of a dedicated bridging stent-graft and the subsequent use of a wide range of “off-label” balloon-expandable stent-grafts selected upon physician’s preference. By the end of 2024, the BeGraft Peripheral stent (BeGraft, Bentley InnoMed) has become the only CE (Conformité Européenne) marked bridging stent-graft available for FEVAR procedures [10]. However, more recently, VIABAHN VBX Balloon-Expandable Endoprosthesis (WL Gore and Associates, Flagstaff, AZ, USA) received CE mark for use as a bridging stent-graft in FEVAR procedures as well.

Independently of the selected bridging stent-graft, the common practice during a FEVAR procedure includes the “flaring” technique of the protruding segment of the stent-graft, with a short 20 mm non-compliant balloon, usually with 8- or 10-mm diameter for the renal artery stent-grafts, and 10- or 12-mm diameter for the visceral stent-grafts [11] However, the “flaring” technique involves additional endovascular maneuvers, including withdrawal of the stent-graft catheter and advancement and inflation of a larger balloon. These extra steps are associated with prolonged fluoroscopy and operative time, and they carry potential risks such as stent-graft dislodgment, loss of the guidewire, as well as failure to advance the larger balloon and subsequently skipping the flaring step.

To address this issue, Bentley InnoMed GmbH (Hechingen, Germany) developed the BeFlared Stent-Graft, which was introduced to the market in November 2024 [10]. The stent-graft is mounted on a balloon that features a larger diameter in the proximal end and a smaller diameter in the distal end, enabling both deployment and flaring in one step. Additionally, besides the usual proximal and distal marker, the new BeFlared catheter has an extra third marker (the “fenestration” marker), which should be aligned with the ring of the fenestration during deployment. These design features are expected to contribute to a more standardized, efficient, and time-saving FEVAR procedure [12].

This study aims to present the geometrical analysis and the early single-center clinical experience of the BeFlared Covered Stent-Graft in FEVAR procedures with CMDs or PMEGs.

## Methods

All consecutive patients with cAAAs and TAAAs who underwent either urgent or elective FEVAR (CMD or PMEG), and received at least one BeFlared stent-graft as the primary bridging stent in a TV, were included. Demographics, comorbidities, and clinical data were recorded prospectively in a dedicated database and retrospectively analyzed. Follow-up data were collected from reports of the aortic outpatient clinic.

Complex AAAs were defined as aneurysms that involved the renal and/or visceral arteries, extended up to the celiac artery and included short-neck infrarenal (< 10 mm), juxtarenal, suprarenal, and pararenal [1]. Thoracoabdominal aneurysms were classified according to the Modified Crawford Classification (Safi Classification) [13].

**Fig. 1:**
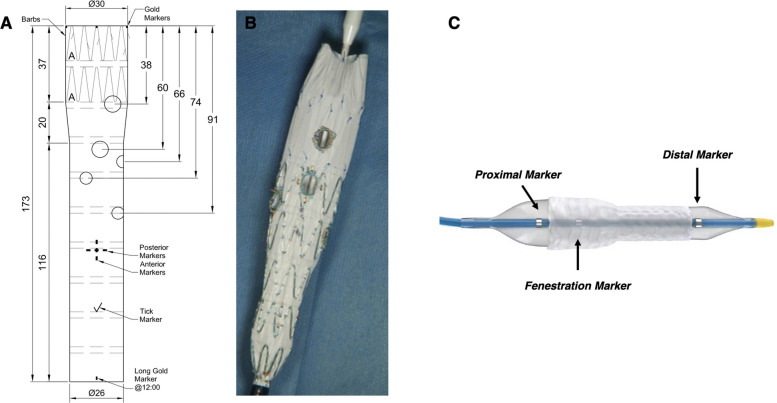
**A** Graft plan of a fenestrated custom-made device (CMD) **B** Physician-modified endograft (PMEG) **C** The BeFlared stent-graft which is mounted on a balloon with different diameters at the proximal and distal part (larger at the proximal end), enabling deployment and flaring of the stent-graft in one step. The arrows demonstrate the three markers of the catheter (proximal, distal, and the “fenestration” marker)

Elective patients routinely underwent repair with custom-made Zenith Fenestrated Endografts (Cook Medical, Bloomington, USA) (Fig. 1A), including a combination of fenestrations and/or branches based on the type of aneurysm and patients’/target vessels’ anatomy. In stable patients undergoing urgent repair (symptomatic, ruptured or large diameter aneurysms), a physician-modified endograft (PMEG) using the Cook platform was deployed (Fig. 1B).

### Stent-graft design

The BeFlared Stent-Grafts are constructed on a cobalt-chromium framework and are coated with a microporous expanded polytetrafluoroethylene (ePTFE) membrane, allowing compatibility with 6Fr catheters up to 6 mm in diameter and 7Fr up to 9 mm in diameter. The catheter is designed to be compatible with 0.035-inch guidewires. The BeFlared stent-graft consists of a BeGraft Peripheral stent-graft mounted on a balloon with different diameters at each end—with a larger diameter proximally. This unique balloon configuration enables simultaneous stent-graft deployment and proximal flaring in a single step, eliminating the need for additional maneuvers to introduce and inflate a separate larger balloon. The proximal portion of the balloon includes an additional radiopaque marker, in addition to the standard proximal and distal markers. This third marker (the “fenestration” marker) provides an important advantage in precise stent positioning, helping to avoid excessive or insufficient protrusion of the stent into the aortic endograft, which is critical for achieving optimal flaring and sealing (Fig. 1C).

### Case planning

All preoperative/postoperative measurements and case-planning were performed on centerline-guided 3D reconstructions on a dedicated workstation (TeraRecon, Emperor Blvd., Durham, NC, USA). The diameter of the TVs was measured at the planned landing zone on a manually adjusted centerline, while the distance of the origin of the TV to its first division branch was analyzed on curved planar reconstructions. To prevent turbulences, but still achieve an adequate fixation in the main body, protrusion of the stent-graft was planned to be approximately 5 to 7 mm, as achieved by alignment of the dedicated “fenestration” marker of the BeFlared stent-graft, while the sealing zone was estimated as a stent-to-vessel wall contact of > 10 mm. Using these parameters, the bridging stent’s diameter and length were preoperatively calculated, with minimal oversizing (< 10%) and no intended coverage of divisional branches. Following its introduction at our institution, the BeFlared stent-graft was adopted as the default bridging device for all TVs requiring fenestration. Use of an alternative stent-graft occurred only in cases where the BeFlared was not available at the time of the procedure. Perioperative data, including procedure duration, fluoroscopy time, volume of contrast agent, and periprocedural events were collected.

Immediately after the procedure, patients received oral dual antiplatelet therapy: aspirin, 100 mg alongside clopidogrel 75 mg, unless they had an underlying condition requiring anticoagulation [14, 15].

### Follow-up

Computed tomography (CT) was performed either prior to discharge or within the first 30 days following the procedure. The postoperative regimen included dual antiplatelet therapy (DAPT) for 6 months, followed by lifelong single antiplatelet therapy in cases of uncomplicated course. In patients requiring additional anticoagulation (e.g., vitamin K antagonists or direct oral anticoagulants), only single antiplatelet therapy was administered. Patients were scheduled for clinical and radiological follow-up at our aortic outpatient clinic at 4 to 6 weeks, 6 months, 12 months, and annually thereafter. Early outcomes were defined as events occurring within 30 days post-procedure. During follow-up, data were collected on major adverse events, target vessel (TV) instability, and mortality.

### Endpoints and definitions

The primary objective of this study was to evaluate the early clinical and technical outcomes of the BeFlared used as a bridging stent-graft for FEVAR procedures. The primary endpoint was the TV-related technical success which was defined as the successful BeFlared insertion, accurate placement ensuring the “fenestration” marker was aligned with the fenestration, and successful stent-graft deployment, with absence of TV-related endoleak on completion angiography (type Ic/type IIIc) and no need for secondary inflation. Secondary endpoints were freedom from target vessel instability (TVI), TV-related reinterventions, TV-related mortality, 30-day and in-hospital mortality, and geometrical layout analysis of the BeFlared. Freedom from TVI was defined as absence of TV or stent-graft occlusion, stenosis, and/or endoleak type Ic/IIIc during follow-up [16].

**Fig. 2:**
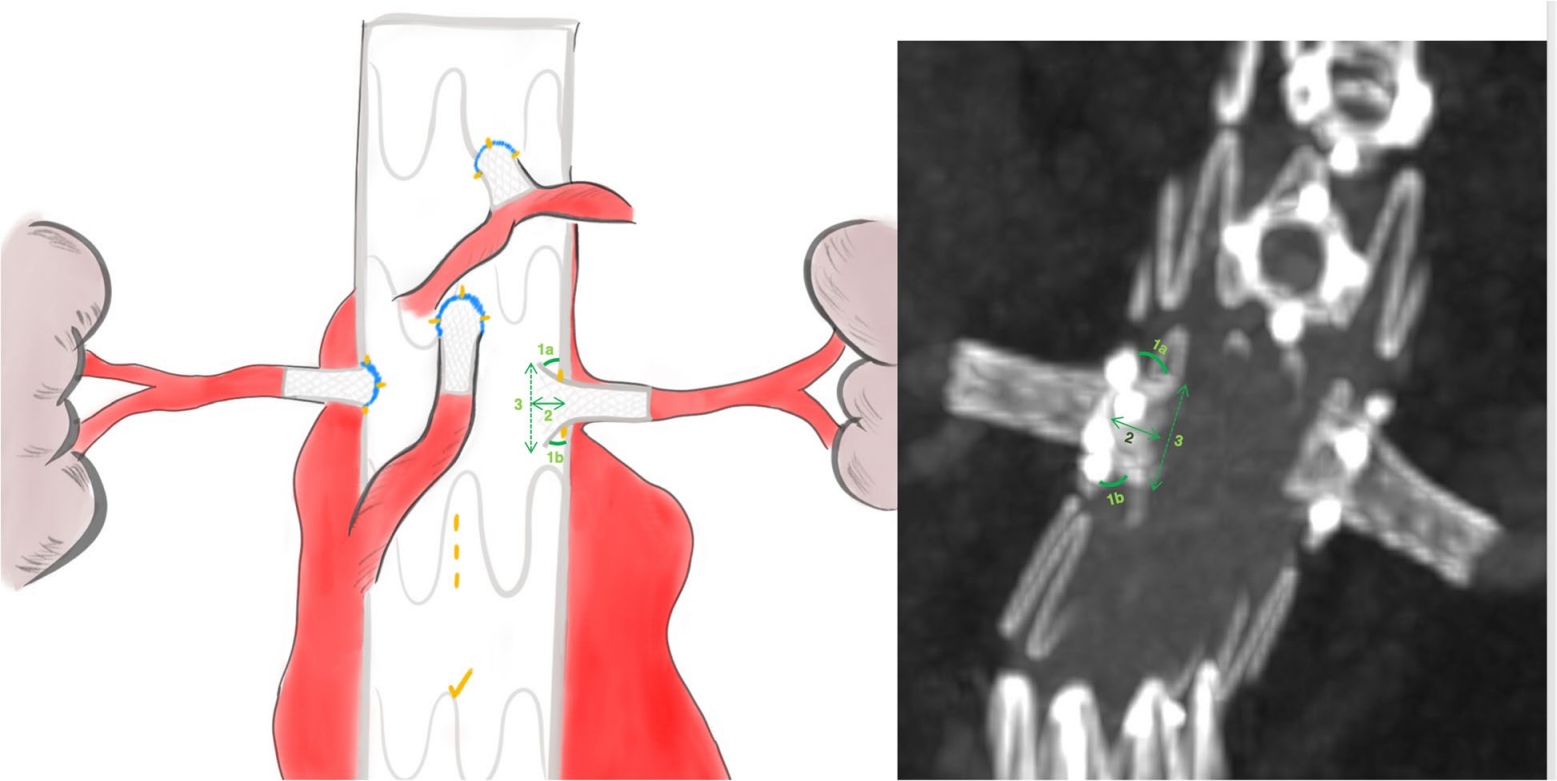
Geometrical analysis of the BeFlared stent-graft: 1a superior flaring angle, 1b inferior flaring angle, 2 protrusion length of the stent-graft inside the endograft lumen, 3 proximal stent-graft diameter which was then divided to the intended stent-graft proximal diameter to calculate the flare ratio). The opening flaring angle was calculated as 180° minus the sum of the superior and inferior flaring angles

**Fig. 3:**
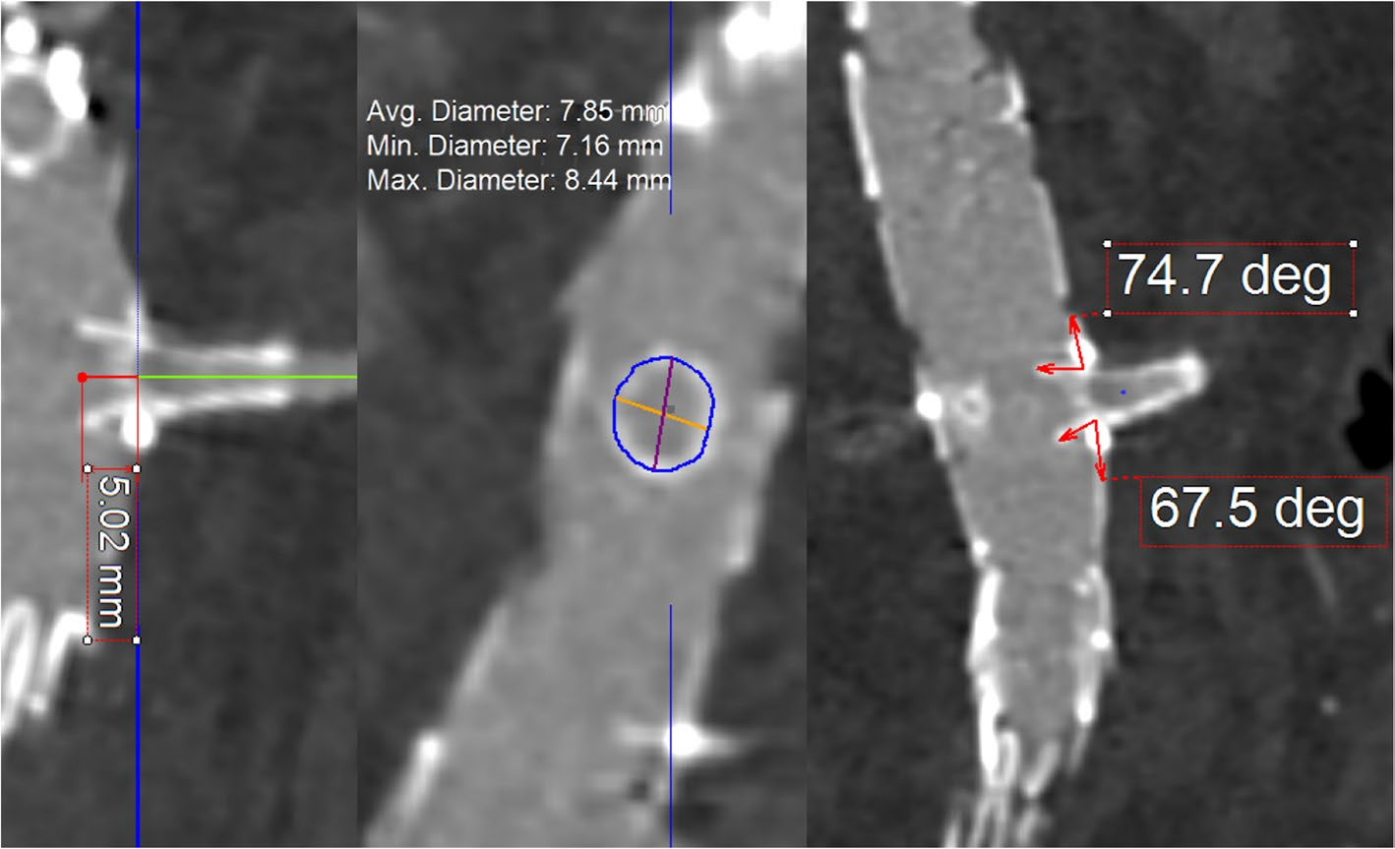
Geometrical analysis of the BeFlared stent-graft, measuring protrusion length of the stent-graft inside the endograft lumen, proximal stent-graft diameter and superior/inferior flaring angle

BeFlared geometrical layout analysis included stent-graft protrusion length inside the endograft lumen, superior and inferior flaring angles, and the opening flaring angle. Opening flaring angle was calculated as 180–(superior flaring angle + inferior flaring angle) [12]. Additionally, the flare ratio was calculated by dividing the proximal stent-graft diameter by the intended stent-graft proximal diameter. For this purpose, the average diameter of the flared proximal part of the BeFlared stent-graft was measured and then divided by the intended proximal flared diameter (e.g., 8 for the BeFlared 6–8 × 27 mm, 10 for the BeFlared 7–10 × 27 mm, etc.). All length measurements were performed using centerline reconstructions, and the flaring angles were measured in vertical multiplanar reformation views centered on the fenestration. Geometrical measurements were performed as illustrated in Figs. 2 and 3.

### Statistical analysis

Statistical data analysis was performed with SPSS Statistics (version 28; IBM, Chicago, IL, USA). Continuous variables are expressed as mean ± standard deviation or median with interquartile ranges, according to the normality of distribution. Categorical variables are presented as number and percentages. For comparison of anatomical parameters, TVs were grouped into visceral and renal TVs. Cross-tables and *t* tests for independent variables were performed to evaluate the difference between renal and visceral stent-graft configuration.

## Results

A total of 24 consecutive patients were treated for cAAA or TAAA between December 2024 and June 2025, each with at least one TV requiring fenestration. Among them, 23 patients (19 males, 82.6%) received at least one BeFlared stent-graft as a bridging stent. The mean age was 72.5 ± 7.9 years, and the median aneurysm diameter was 60 mm (54–66). The indication for treatment included cAAA (*n* = 15, 65.2%) and TAAA (*n* = 8, 34.8%). The majority of patients (*n* = 16, 69.6%) were treated electively, and the remaining were performed in an urgent setting including 6 patients (26.1%) with symptomatic aneurysms and one patient (4.3%) with a ruptured aneurysm. Of the 23 patients, 16 (69.6%) underwent repair with custom-made Cook stent-grafts (Cook Medical, Bloomington, USA) (Fig. 1B). The remaining 7 patients (30.4%) were treated with physician-modified endografts using the Cook platform (Thoracic Alpha or TX2) (Fig. 1B, C). Within the CMD cohort, three patients had an in-built bifurcated device, whereas all other cases required a separate distal bifurcated component. Patients’ demographics and characteristics are shown in Table 1.

**Table 1:** Patients’ demographics and characteristics

	*N* = 23 (%)
Male gender	19 (82.6)
Age, years	72.5 ± 7.9
BMI (kg/m^2^)	24.7 ± 4.6
Hypertension	19 (82.6)
Dyslipidemia	14 (60.9)
Diabetes mellitus	4 (17.4)
COPD	4 (17.4)
CAD	9 (39.1)
Chronic heart failure	2 (8.7)
Past Stroke/TIA	2 (8.7)
Smoker (current or ex)	7 (30.4)
ASA class ≥ III	21 (91.3)
III	17 (73.9)
IV	4 (17.4)
Previous Aortic Surgery	7 (30.4)
Ascending/arch repair	2 (8.7)
TEVAR	7 (30.4)
EVAR	2 (8.7)
Abdominal aortic surgery	0 (0)
Complex AAA	15
Juxtarenal	11
Pararenal	4
TAAA	8
Type I	2
Type II	1
Type III	2
Type IV	1
Type V	2
Pre-operative diameter (mm, median, IQR)	60 (54—66)
Elective	16 (69.6%)
Urgent	7 (30.4%)
Symptomatic	6 (26.1%)
Rupture	1 (4.3%)
CMD	16 (69.6%)
PMEGs	7 (30.4%)

A total of 70 TVs was bridged with at least one BeFlared stent-graft: 30 visceral TVs (11 in the celiac trunk [CT] and 19 in the superior mesenteric artery [SMA]) and 40 renal TVs (18 right renal arteries [RRAs], 19 left renal arteries [LRAs], and 3 accessory renal arteries). The distal stent-graft diameter was 5 mm in 8 TVs (11.4%), 6 mm in 20 TVs (28.6%), 7 mm in 21 TVs (30%), 8 mm in 8 TVs (11.4%), and 9 mm in 13 TVs (18.6%). In the majority of cases (41 TVs, 58.6%), a 27 mm-long stent-graft was used. A 22 mm-long stent-graft was deployed in 28 TVs (40%), and in one case (1.4%), a 37 mm-long stent-graft was selected (Table 2).

**Table 2:** Target vessels and stent-graft configuration

	*N* = 70 (%)
Visceral Arteries	30 (42.8)
Celiac Trunk	11 (15.7)
Superior Mesenteric Artery	19 (27.1)
Renal Arteries	40 (57.1)
Right Renal Artery	18 (25.7)
Left Renal Artery	19 (27.1)
Accessory Renal Artery	3 (4.3)
Stent-Graft Diameter	
5–8 mm	8 (11.4)
6–8 mm	20 (28.6)
7–10 mm	21 (30)
8–10 mm	8 (11.4)
9–11 mm	13 (18.6)
Stent-Graft Length	
22 mm	28 (40)
27 mm	41 (58.6)
37 mm	1 (1.4)

TV-related technical success was 100% (70/70). In all cases, the insertion of the BeFlared stent-graft was successful, without technical difficulties. The placement of the “fenestration marker” was accurate and well-aligned with the fenestration, resulting in successful stent-graft deployment in all 70 target vessels (TVs). There were no cases requiring secondary balloon inflation with a larger balloon due to inadequate flaring. Both TV selective angiography and completion angiography showed no evidence of TV-related endoleak (Type Ic, Type IIIb or Type IIIc).

No perioperative adverse events on integrity of bridging stents were observed during and after advancement of distal bifurcated component or iliac limbs. Perioperative data are shown in Table 3.

**Table 3:** Peri-operative and 30-Day Results

	*N* = 23(%)	
Total operation time – (minutes, median, IQR)	182 (160–242)	
Total fluoroscopy time – (minutes, median, IQR)		46.5 (39–68.2)
Contrast dose (mL, median, IQR)	165 (105–270)	
Technical Success	23 (100)	
30-day overall Mortality	1 (4.3)	
30-day overall Reintervention	1 (4.3)	
TV-related 30-day outcomes		
Technical Success	70 (100)	
Target Vessel Instability	0 (0)	
Re-intervention	0 (0)	

### Thirty-day outcomes

Freedom from target vessel (TV) instability was 100% (70/70), and no TV-related reinterventions were reported, while TV-related 30-day mortality was 0%. No TV-related endoleak (Type Ic or Type IIIc) and/or TV occlusion was detected on 30-day CTA follow-up. The overall reintervention rate was 4.3% (1/23), and the all-cause 30-day mortality was also 4.3% (1/23). Both events occurred in the same patient, who initially presented with a ruptured aneurysm. This patient underwent a non-TV-related reintervention for a concomitant aortogastric fistula and subsequently died from multiorgan failure. The complete 30-day outcomes are summarized in Table 3.

### BeFlared geometrical layout analysis

A total of 70 implanted BeFlared stent-grafts were analyzed for geometrical layout analysis (Table 4). The mean protrusion length of the stent-graft into the endograft lumen was 5.27 mm ± 0.87, while the mean superior and inferior flaring angles were 67.21^o^ ± 11.3 and 70.95^o^ ± 15.7, respectively. The mean flare ratio of the proximal stent-graft diameter to the indented proximal diameter was 0.96 ± 0.03. The mean opening flaring angle was 41.8^o^ ± 19.1.

**Table 4:** Geometrical Layout BeFlared Analysis

	Overall (*n *= 70)		Visceral BeFlared (*n* = 30)		Renal BeFlared (*n* = 40)	*p*
Protrusion Length (mm)	5.27 ±.87			5.1 ±.18	5.4 ±.12	.155
Superior flaring angle (^o^)	67.2 ± 11.3			63.6 ± 11.30	69.8 ± 10.66	.022
Inferior flaring angle (^o^)	70.9 ± 15.7			76.1 ± 17.20	67.8 ± 13.98	.054
Opening flaring angle (^o^)	41.8 ± 19.1			41.2 ± 19.4	42.3 ± 19.1	.809
Flare ratio	0.96 ±.03			0.95 ±.03	0.97 ±.03	.045

In a subgroup analysis comparing renal and visceral BeFlared stent-grafts, the mean protrusion length was 5.4 ± 0.12 mm for renal stent-grafts and 5.1 ± 0.18 mm for visceral stent-grafts (*p* = 0.155). The mean flare ratio was slightly higher in renal stent-grafts (0.97 ± 0.03) compared to visceral stent-grafts (0.95 ± 0.03), with a statistically significant difference (*p* = 0.045). The mean opening flaring angle was 42.3° ± 19.1 for renal stent-grafts and 41.2° ± 19.4 for visceral stent-grafts (*p* = 0.809).

## Discussion

This study presents a single-center evaluation of the BeFlared bridging stent-graft used in FEVAR procedures, focusing on both postoperative geometrical configuration and early clinical outcomes. A total of 70 TVs was treated with a 100% technical success rate, and no TV-related endoleak, reintervention, or occlusion was observed at 30 days. The mean protrusion length was 5.27 ± 0.87 mm, and the flare ratio was 0.96 ± 0.03, demonstrating consistent flaring performance across renal and visceral arteries.

Several studies have previously evaluated the clinical performance of the BeGraft peripheral stent-graft, which forms the base platform of the BeFlared system. In a multicenter study by Clough et al. [17], 101 TVs treated with BeGraft bridging stent-grafts showed a patency rate of 97% at a median of 33 months, with freedom from TVI, reintervention, and overall survival of 95%, 82%, and 72%, respectively. Similarly, D’Oria et al. [18] analyzed 266 BeGraft-bridged TVs and reported a technical success rate of 94% and freedom from TVI of 97.9% and 97.2% at 12 and 24 months, respectively. Prior data from our own institution involving 361 TVs treated with BeGraft peripheral reported a technical success rate of 99.4% and a cumulative TVI rate of 0.8% during a median 20-month follow-up, with 1-, 2-, and 3-year freedom from TVI exceeding 98% [19]. In the present study, the early clinical outcomes of the BeFlared stent-graft appear consistent with these earlier BeGraft peripheral results. Technical success was achieved in 100% of the 70 treated TVs, with no target vessel instability or reinterventions recorded at 30-day follow-up.

The early safety and efficacy of the BeFlared bridging stent-graft are further supported by the recent prospective multicenter BeSafe study, which included 84 patients across 11 high-volume centers. In this cohort, TV catheterization failed in only 2 of 312 vessels (0.6%), and BeFlared deployment was successful in all remaining cases (*n* = 310) [20]. Procedural performance was favorable, with a mean operative time of 162 ± 60 min, low need for secondary balloon inflation (4.2%), and additional bridging stents required in only 1.9% of TVs. Completion angiography and postoperative CTA demonstrated excellent early results, with just one type IC endoleak (0.3%) and one renal stenosis (0.3%), leading to a 99.3% freedom from TVI at 1 month and no cases of stent kinking, fracture, or occlusion. Importantly, geometric analysis of 300 implanted BeFlared stents showed consistent deployment, with small fenestration-to-ostium gap distances (1.1–1.7 mm across visceral and renal vessels), a mean protrusion length of 5.4 ± 1.4 mm, and mean superior and inferior flaring angles of 72.3° ± 14.0 and 77.4° ± 15.8°, respectively [20].

These findings are consistent with the results reported by Veraldi et al. [21], where the use of BeFlared in 31 target vessels significantly improved procedural efficiency compared with the BeGraft peripheral cohort of 185 TVs. BeFlared reduced operative time (241 vs 270.3 min; *p* = 0.022), shortened bridging stent deployment time (5 vs 7.6 min; *p* < 0.001), and showed a trend toward reduced fluoroscopy duration. Geometric assessment further demonstrated more uniform deployment, with reduced variability in TV landing zone (16.4 vs 15.4 mm; *p* < 0.001), shorter aortic protrusion (4.2 vs 5.1 mm; *p* < 0.001), and larger proximal diameters and flaring angles [21].

Likewise, Pennetta et al. [22] found significant procedural benefits in a controlled comparison of 60 patients (240 target vessels), where BeFlared achieved shorter stenting times (11.3 min; IQR 10.9–12.0 vs 17.4 and 20.2 min; *p* < 0.001), reduced radiation exposure (dose area product 4.6 vs 9.3 and 11.4 Gy·cm^2^; *p* = 0.001) and shorter fluoroscopy times for all TVs (*p* < 0.001). Postoperative imaging also demonstrated larger flaring diameters across most TVs (celiac *p* = 0.002; SMA *p* = 0.002; right renal *p* < 0.001; left renal *p* = 0.126), indicating a better flaring ability [22]. Preclinical data by Oliny et al. [12] compared the BeFlared and BeGraft devices using perfused 3D-printed patient anatomies. Both devices achieved 100% assisted primary efficacy, but BeFlared demonstrated significantly larger proximal flaring diameters (10.5 mm vs. 9.2 mm; *p* < 0.001), smaller superior flaring angles (77.6° vs. 84.7°; *p* = 0.005), and a greater opening angle (46.1° vs. 33.4°; *p* < 0.001), suggesting more effective proximal expansion due to the integrated stepped balloon [12].

Together, these external data align closely with our geometric analysis, which demonstrated uniform BeFlared deployment characterized by consistent protrusion length (5.27 ± 0.87 mm), high flare ratios, and stable early outcomes without TVI. The converging evidence supports the notion that the dedicated design enhances procedural efficiency and contributes to more standardized stent-graft geometry, with potential implications for long-term durability.

Most studies evaluating flaring performance have used the flare-to-fenestration diameter ratio, which is the ratio of the proximal stent diameter to the diameter of the corresponding fenestration. However, this approach can be misleading, as fenestration dimensions (e.g., 6 × 6 mm or 6 × 8 mm for renal arteries) vary between operators and institutions, introducing variability unrelated to the stent’s true flaring behavior. In our study, we instead calculated the flare ratio as the ratio of the measured proximal stent diameter to the intended proximal diameter based on the device specification (e.g., 8 mm for a 6–8 mm BeFlared). This method could offer several advantages. First, it provides a device-centered performance metric, directly assessing whether the stent achieved its intended expansion rather than how it fits within a variable fenestration. Additionally, it improves inter-study comparability, enabling consistent evaluation across centers and studies using the same stent-graft. Finally, it is more sensitive to under‑ or over-deployment, since deviations from the nominal flare ratio immediately reflect suboptimal expansion. Together, these factors make the flare ratio a more reliable and reproducible indicator of actual device performance.

Using this method, we observed a mean flare ratio of 0.96 ± 0.03, indicating effective and consistent flaring across target vessels. Renal stent-grafts had a slightly higher flare ratio than visceral ones (0.972 vs. 0.957; *p* = 0.045), though the absolute difference was modest. This approach allowed for clearer interpretation of flaring performance across patients and subgroups, minimizing confounding by operator-specific planning preferences.

Several studies have also analyzed the flaring performance of other bridging stents such as VBX and Advanta V12. For example, Fouad et al. [23] reported a mean flare ratio of 1.19 ± 0.17 and flare diameter of 8.5 ± 1.4 mm for VBX, with flare-to-fenestration distances ranging from 4.5 to 6.8 mm. Overeem et al. [24] found a mean flare ratio of 1.14 ± 0.19 among 31 bridging stents, primarily VBX and Advanta. In a larger analysis by Squizzato et al. [25], VBX stents demonstrated a higher postflare diameter (8.7 ± 1.9 mm), greater flare ratio (1.27 ± 0.27), and shorter protrusion length (5.7 ± 2.2 mm) compared to a mixed group of other stents including BeGraft. Notably, temporal selection bias and evolving operator experience were acknowledged as potential confounding factors in that analysis.

Taken together, these comparisons suggest that the BeFlared bridging stent-graft delivers consistent and reproducible geometrical outcomes, in line with both its BeGraft predecessor and other commonly used bridging stent platforms. While differences in flare ratios, protrusion lengths, and diameters may reflect device-specific design as well as operator technique, early clinical outcomes across platforms appear broadly comparable. Long-term follow-up and standardized geometric assessment protocols will be necessary to determine whether these differences translate into clinically meaningful advantages in freedom from TVI.

## Limitations

The limitations of this study include its retrospective design and single-center setting. Additionally, as the geometrical analysis was based solely on 1-month follow-up CTA, the durability of the favorable technical results could not be evaluated. An additional limitation is that the geometric analysis was based on manual measurements and is therefore subject to a certain degree of measurement error. Finally, the small sample size of 70 BeFlared stent-grafts, the absence of a control group, and the lack of an event of TVI observed in this cohort represent additional limitations of the study.

## Conclusion

The data from this study underline the promising early-term clinical outcomes of the BeFlared stent-graft as a bridging stent in FEVAR, along with satisfactory technical performance. Follow-up studies reporting mid- and long-term outcomes are essential to assess the durability of the BeFlared stent-graft.

## Data Availability

The datasets generated and/or analyzed during the current study are available from the corresponding author upon reasonable request.
